# Arsenic Transport in Rice and Biological Solutions to Reduce Arsenic Risk from Rice

**DOI:** 10.3389/fpls.2017.00268

**Published:** 2017-03-01

**Authors:** Yanshan Chen, Yong-He Han, Yue Cao, Yong-Guan Zhu, Bala Rathinasabapathi, Lena Q. Ma

**Affiliations:** ^1^State Key Laboratory of Pollution Control and Resource Reuse, School of the Environment, Nanjing University Nanjing, China; ^2^Key Laboratory of Urban Environment and Health, Institute of Urban Environment, Chinese Academy of Sciences Xiamen, China; ^3^Horticultural Sciences Department, University of Florida, Gainesville FL, USA; ^4^Soil and Water Science Department, University of Florida, Gainesville FL, USA

**Keywords:** *Oryza sativa*, arsenic uptake, arsenate reduction, sequestration, arsenite efflux, methylation

## Abstract

Rice (*Oryza sativa* L.) feeds ∼3 billion people. Due to the wide occurrence of arsenic (As) pollution in paddy soils and its efficient plant uptake, As in rice grains presents health risks. Genetic manipulation may offer an effective approach to reduce As accumulation in rice grains. The genetics of As uptake and metabolism have been elucidated and target genes have been identified for genetic engineering to reduce As accumulation in grains. Key processes controlling As in grains include As uptake, arsenite (AsIII) efflux, arsenate (AsV) reduction and AsIII sequestration, and As methylation and volatilization. Recent advances, including characterization of AsV uptake transporter OsPT8, AsV reductase OsHAC1;1 and OsHAC1;2, rice glutaredoxins, and rice ABC transporter OsABCC1, make many possibilities to develop low-arsenic rice.

## Arsenic In Rice

Rice (*Oryza sativa* L.), the staple food for half of the world’s population, is widely cultivated. An estimated 741 million tons valued at US $191 billion are produced annually (2013 data from the FAO^1^). However, rice also has a negative side. Besides taking up heavy metals like cadmium, it is an efficient accumulator of arsenic (As), a toxic metalloid, making rice consumption a major source of As exposure to humans (Sohn, 2014).

Arsenic is ubiquitous in the environment and its contamination in soil and water has been reported in many countries. In aerobic soils, As is mainly present in the oxidized form as arsenate (AsV). While in anaerobic environments like paddy soil, it mainly exists in the reduced form as arsenite (AsIII) (Huang et al., 2011). Compared to other cereals, rice is more efficient in accumulating As in its grains (Williams et al., 2007; Sohn, 2014). This is because rice is often grown in flooded soils where more mobile AsIII is the dominant form in submerged environment (Xu et al., 2008). In addition, rice is a silicon (Si) accumulating plant and requires large amounts of Si for optimal growth, making up to 10% of the shoot biomass (Ma and Yamaji, 2006). The efficient Si uptake pathway in rice also allows inadvertent passage of AsIII due to their chemical similarity. They both exist as neutral species in paddy soils, i.e., arsenous acid-As(OH)_3_ and silicic acid-Si(OH)_4_ (Ma et al., 2008).

Arsenic exposure through drinking water adversely impacts millions of people, leading to increased cancer risk (Argos et al., 2010; Joseph et al., 2015). In Bangladesh, As-contaminated groundwater has been widely used to irrigate rice, leading to high As in Bangladeshi rice (Williams et al., 2006). The situation is also urgent in many other Asian regions including India, Vietnam, Cambodia, Thailand, and China with As-contaminated soils and water where rice is a national staple (Li G. et al., 2011; Rahman and Hasegawa, 2011). In addition, rice is consumed all over the world including the US and Europe, making As in rice a global issue of concern (Zhu et al., 2008; Meharg et al., 2009; Gilbert-Diamond et al., 2011).

The As in rice grain is present primarily as inorganic AsIII and AsV, with a considerable proportion (typically 20–50%) of organic As, mainly as dimethylarsinic acid (DMA^V^) (Williams et al., 2005). However, it has been reported that rice is unable to methylate inorganic As *in vivo*, thus methylated As species most likely come from the rhizosphere via microbial methylation (Lomax et al., 2012; Jia et al., 2013).

Research has uncovered the physiology of how plants deal with As. While arsenic accumulation in rice can be reduced by modifying cultural practices (Wang et al., 2015), this review focuses on the genetic solutions for developing varieties with low As accumulation ability. Given the magnitude of the problem and the vast number of people being affected, there is an urgent need to produce rice with low As. In this context, gene modification is an effective and practical approach to reduce As accumulation in rice grains. This approach taps into the potential of various genes controlling As uptake, transformation and translocation in plants. Some genes have been proven to affect As accumulation in transgenic plants while others need further research (**Table 1**). Here, we review the genes controlling As metabolism, describe recent progress in producing low-As rice, and discuss the potential utilization of CRISPR/Cas9-based genome-editing technology to reduce As uptake, translocation and accumulation, thereby lowering the As content in rice grain.

**Table 1:** Critical gene families and representative genes from different species involved in As uptake, transport and metabolism.

Gene category	Gene name	Source	Manipulation	Consequence	Reference
Phosphate transporter (AsV transport)	*AtPht1;1/4*	*A. thaliana*	Knockout	Increased AsV tolerance	Shin et al., 2004
	*OsPht1;8* (*OsPT8*)	*O. sativa*	Knockout	Decreased AsV uptake; Increased AsV tolerance	Wang et al., 2016
Aquaporins (AsIII transport)	*Lsi1* (*OsNIP2;1*)	*O. sativa*	Knockout	Decreased As accumulation in straw of field-grown rice	Ma et al., 2008
	*AtNIP1;1*	*A. thaliana*	Knockout	Increased AsIII tolerance; Decreased As accumulation	Kamiya et al., 2009
	*AtNIP3;1*	*A. thaliana*	Knockout	Increased shoot As tolerance; Decreased shoot As	Xu et al., 2015
	*PvTIP4;1*	*P. vittata*	Overexpression (*Arabidopsis*)	AsIII sensitivity; Increased As accumulation	He et al., 2016
Arsenate reductase	*AtACR2*	*A. thaliana*	Knockout or overexpression	No effect on As accumulation	Liu et al., 2012
	*AtHAC1*	*A. thaliana*	Knockout	AsV sensitivity; Decreased As efflux from roots; Increased As accumulation in the shoots	Chao et al., 2014; Sanchez-Bermejo et al., 2014
	*OsHAC1;1 & OsHAC1;2*	*O. sativa*	Overexpression (rice)	Increased AsIII efflux into the external medium; Decrease As accumulation in rice grain	Shi et al., 2016
Glutaredoxin	*PvGrx5*	*P. vittata*	Overexpression (*Arabidopsis*)	Increased As tolerance; Decreased As in leaves	Sundaram et al., 2009
	*OsGrx_C7 & OsGrx_C2.1*	*O. sativa*	Overexpression (*Arabidopsis*)	Increased As tolerance; Decreased As accumulation	Verma et al., 2016
Phytochelatin synthase	*CdPCS1*	*C. demersum*	Overexpression (rice)	Decreased As accumulation in grain	Shri et al., 2014
NRAMP transporter (Fe/Mn/Cd/As transport)	*OsNRAMP1*	*O. sativa*	Overexpression (rice)	Increased As tolerance and accumulation	Tiwari et al., 2014
ABC transporter (Cd/Pb/As transport)	*YCF1*	*S. cerevisiae*	Overexpression (*Arabidopsis*)	Increased As tolerance and accumulation	Song et al., 2003; Guo et al., 2012
	*AtABCC1/2*	*A. thaliana*	Overexpression (*Arabidopsis*)	Increased As tolerance	Song et al., 2010
	*OsABCC1*	*O. sativa*	Overexpression (*Arabidopsis*)	Increased As tolerance	Song et al., 2014
ACR3 transporter (AsIII efflux)	*ScACR3*	*S. cerevisiae*	Overexpression (rice)	Increased As efflux; Decreased As in grain	Duan et al., 2012
	*PvACR3*	*P. vittata*	Overexpression (*Arabidopsis*)	Increased As efflux; Decreased As accumulation under AsIII in short-term exposure; Increased shoot As accumulation in soil in long-term cultivation	Chen Y. et al., 2013
ArsB/NhaD permease (AsIII efflux)	*ArsB*	*E. coli*	Knockout	As sensitivity and As accumulation	Meng et al., 2004
	*Lsi2*	*O. sativa*	Knockout	Decreased As accumulation	Ma et al., 2008
ArsM/AS3MT family (As methylation)	*RpArsM*	*R. palustris*	Overexpression (rice)	Produced methylated volatile arsenic	Qin et al., 2006; Meng et al., 2011
	*CmArsM7/8*	*C. merolae*	Expression (*E. coli*)	Conferred resistance to AsIII	Qin et al., 2009
	*CrarsM*	*C. reinhardtii*	Overexpression (*Arabidopsis*)	As methylation to DMA^V^ and As sensitivity	Tang et al., 2016
Inositol transporters (As transport)	*AtINT2/4*	*A. thaliana*	Knockout	Lower shoot As accumulation	Duan et al., 2015
CRT-like transporter (Glutathione homeostasis)	*OsCLT1*	*O. sativa*	Knockout	Lower As accumulation in roots but higher or similar As accumulation in shoots	Yang et al., 2016


## Arsenic Metabolism in Rice

### As Uptake in Rice

**FIGURE 1:**
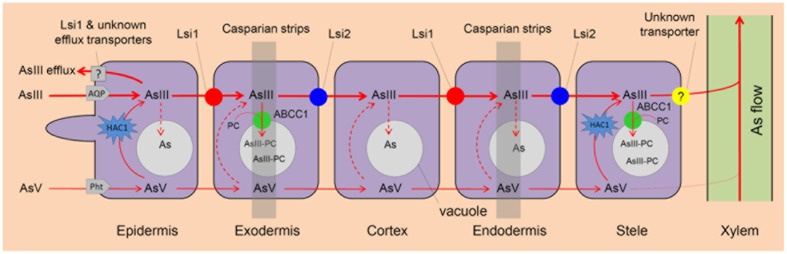
**Arsenite (AsIII) and arsenate (AsV) uptake and As vacuolar sequestration in rice roots.** AsIII and AsV are taken up by rice roots via aquaporins (AQP) and phosphate transporters (Pht), respectively. AsV is reduced to AsIII by arsenate reductase HAC1. AsIII influx transporter Lsi1 and AsIII efflux transporter Lsi2 play a critical role in As uptake and As transport to root xylem for translocation respectively. AsIII can be chelated by phytochelatins (PC) and ABCC1 is an AsIII-PC complex transporter that localizes to the vacuolar membrane and transport As into root vacuoles for sequestration. Lsi1 and other unknown transporters mediate AsIII efflux. An unknown transporter is involved in AsIII xylem loading.

Arsenate is the main As species in aerobic soils but it accounts for a small amount of total As in flooded paddy soils (Huang et al., 2011; Jia et al., 2014). Rhizospheric processes, such as oxygen release by rice roots, iron plaque formation, and microbial oxidation, all contribute to AsIII oxidation to AsV in soils (Liu et al., 2006; Jia et al., 2014). As a phosphate analog, AsV is taken up by phosphate transporters, including AtPht1;1/4 in *Arabidopsis* (Shin et al., 2004), PvPht1;3 in *Pteris vittata* (DiTusa et al., 2016) and OsPht1;8 (OsPT8) in rice (Wu et al., 2011; Wang et al., 2016). Knockout of OsPht1;8 decreases AsV uptake by 33–57% and significantly increases AsV tolerance in rice (Wang et al., 2016). Following uptake, AsV can be rapidly reduced to AsIII in plant cells by the newly identified HAC1 (High Arsenic Content 1) arsenate reductases (Shi et al., 2016) (**Figure 1**).

Arsenite, a neutral molecule, is the dominant As species in flooded paddy soils (Zhao et al., 2009). It enters plants via aquaporin channels, mainly the nodulin 26-like intrinsic proteins (NIPs, a subfamily of the aquaporin family) (Ma et al., 2008; Kamiya et al., 2009; Mitani-Ueno et al., 2011; Xu et al., 2015) (**Table 1**). In *Arabidopsis*, aquaporins like NIP1;1 and NIP3;1 play an important role in AsIII uptake and translocation (Kamiya et al., 2009; Xu et al., 2015). In rice, the Si influx transporter Lsi1 (Low silicon rice 1; OsNIP2;1) is responsible for AsIII uptake while Si efflux transporter Lsi2 (Low silicon rice 2) mediates AsIII efflux (Ma et al., 2006, 2007, 2008). Both Lsi1 and Lsi2 localize at the plasma membrane of exodermal and endodermal cells of the roots, but with different polar localization, i.e., Lsi1 protein localizes at the distal side of the cell while Lsi2 at the proximal side (Ma et al., 2006, 2007) (**Figure 1**). Thus, the concerted function of these two produces an effective flow of both Si and AsIII across the endodermis and toward the xylem for their translocation (Ma et al., 2008; Zhao et al., 2009) (**Figure 1**). Knockout mutant *lsi1* shows lower As concentrations in the straw, but no significant difference in the grain. In contrast, the *lsi2* knockout significantly decreases As concentrations in the straw and grain, which are 13–19% and 51–63% of the corresponding wild-type rice respectively (Ma et al., 2008). These results indicate that Lsi2 plays a more critical role than Lsi1 in As transport toward the rice grain but knockout of Lsi2 also disrupts Si uptake, which can inhibit rice growth and decrease the grain yield by 60% (Ma et al., 2007).

Besides Lsi1 (OsNIP2;1), other NIPs including OsNIP1;1, OsNIP2;2, OsNIP3;1, and OsNIP3;2 also show permeability to AsIII (Bienert et al., 2008; Ma et al., 2008). Moreover, some plasma membrane intrinsic proteins (PIPs, another subfamily of the aquaporin family), including OsPIP2;4, OsPIP2;6 and OsPIP2;7, are also involved in AsIII transport (Mosa et al., 2012). In addition, the rice NRAMP (Natural Resistance-Associated Macrophage Protein) transporter, OsNRAMP1, may also be involved in AsIII transport as its expression enhances As accumulation in the roots and shoots of *Arabidopsis* (Tiwari et al., 2014) (**Table 1**). It is also reported that OsNRAMP1 localizes on plasma membrane of endodermis and pericycle cells, and may involve in AsIII xylem loading for root to shoot As translocation (Tiwari et al., 2014). Though OsNRAMP1 has been studied in *Arabidopsis*, its specific function in rice still needs further investigation.

In contrast to the AsIII transporters, Fe plaque plays a role in sequestrating As and reducing As uptake by rice (Wu et al., 2012; Lee et al., 2013). Iron plaque is formed on rice roots through oxidization of Fe^2+^ to Fe^3+^, mainly due to the radial movement of oxygen from the aerenchyma to the soil (radial oxygen loss-ROL) and microbial activities (Colmer, 2003; Mei et al., 2009). As a result of adsorption and/or co-precipitation, Fe plaque can sequester As on rice roots, playing an important role in reducing As uptake and accumulation, potentially alleviating As toxicity (Liu and Zhu, 2005; Ultra et al., 2009). It is reported that root ROL rates, which vary with rice genotypes, control Fe plaque formation (Li H. et al., 2011; Wu et al., 2012). Higher rates of ROL increase Fe plaque formation, providing more As sequestration sites on rice roots (Wu et al., 2012).

A number of methylated As species have been detected in soils, among them, monomethylarsonic acid (MMA^V^) and DMA^V^ are the most common (Zhao et al., 2010b; Huang et al., 2011). Methylated As species in rice grains are likely from soils as rice is unable to methylate As *in vivo* (Lomax et al., 2012; Jia et al., 2013). In flooded paddy soils, organic As can be reduced to volatile arsine, including monomethylarsine (MMA^III^), dimethylarsine (DMA^III^) and trimethylarsine (TMA^III^) (Cullen and Reimer, 1989; Huang et al., 2011; Jia et al., 2013). Methylated As can be taken up by rice, but less efficiently than AsIII and AsV (Abedin et al., 2002). A recent study also shows that the Si and AsIII transporter Lsi1 may mediate the uptake of methylated As in rice (Li et al., 2009). Considering DMA^V^ is ∼100-fold less toxic than AsIII in animal cells, DMA^V^ in the grains may reduce As toxicity in rice (Hirano et al., 2004).

### As Detoxification in Rice

Arsenite has high affinity to sulfhydryl (–SH) groups of peptide thiol such as glutathione (GSH) and phytochelatins (PCs), thus AsIII is detoxified by complexing with GSH or PCs in plants (Pal and Rai, 2010). GSH is synthesized in a two-step pathway catalyzed by the rate-limiting γ-glutamylcysteine (γ-EC) synthetase to synthesize γ-EC, followed by glutathione synthetase to combine Gly and γ-EC (Hell and Bergmann, 1990; Dhankher et al., 2002). PCs are synthesized by the transpeptidation of gamma-glutamylcysteinyl dipeptides from GSH by the catalytic activity of phytochelatin synthase (PCS) (Pal and Rai, 2010; Shri et al., 2014). Overexpression of PCS enhances As tolerance in transgenic plants and may also affect their As accumulation (Liu et al., 2010; Pal and Rai, 2010). Shri et al. (2014) showed that the transgenic rice overexpressing *Ceratophyllum demersum* PCS, CdPCS1, increases As levels in the roots and shoots, but significantly lowers As levels in the grain. More recently, a rice CRT (Chloroquine Resistance Transporter)-like transporter, OsCLT1, was shown to play a role in GSH homeostasis, probably by mediating the export of γ-EC and GSH from plastids to the cytoplasm. Moreover, under As treatment, *Osclt1* mutant exhibits much lower PC_2_ contents compared to wild-type, resulting in lower As accumulation in the roots but higher or similar As accumulation in the shoots (Yang et al., 2016).

In yeast, besides AsIII extrusion, there is a second pathway for As detoxification, i.e., vacuolar sequestration of AsIII by YCF1 (Yeast Cadmium Factor) (Ghosh et al., 1999). As a member of the ABC (ATP binding cassette) transporter family, YCF1 can transport GSH conjugate of Cd [Cd(GS)_2_] and As [As(GS)_3_] into vacuoles for sequestration (Li et al., 1997; Ghosh et al., 1999; Song et al., 2003) (**Table 1**). In plants, after being chelated by PC, As can also be sequestrated into vacuoles, which is mediated by C-type ABC transporters (ABCC) (Briat, 2010; Song et al., 2010, 2014). In *Arabidopsis*, AtABCC1 and AtABCC2 mediate AsIII–PC complex transport to the vacuole and overexpression of AtABCC1 increases As tolerance only when co-expressed with PCS, indicating the cooperation of PC synthesis and AsIII–PC complex transporters in plant As detoxification (Song et al., 2010). In rice, a similar ABC transporter, OsABCC1, is critical for the vacuolar AsIII–PC sequestration and As detoxification, so knockout of OsABCC1 leads to increased As sensitivity (Song et al., 2014).

### As Transport in Rice Nodes

Rice nodes are important hubs for controlling its elemental distribution (Yamaji and Ma, 2014; Chen Y. et al., 2015). It has been reported that, with much higher As concentrations than internodes and leaves, rice nodes are the most crucial place for As storage, serving as a filter restricting As transfer to the shoots and rice grains (Song et al., 2014; Yamaji and Ma, 2014; Chen Y. et al., 2015). At the reproductive stage, OsABCC1 is expressed in vascular tissues like the uppermost node I and limits As transport to the grains by sequestering As in the phloem companion cells (Song et al., 2014). Besides, Lsi2 also shows high-level expression in node I where Lsi2 enhances Si distribution into rice panicle, but unfortunately also helps AsIII transport to rice grains (Ma et al., 2008; Yamaji et al., 2015).

A study on As unloading into rice grain shows that DMA^V^ is translocated to the rice grain with over 10 times greater efficiency than inorganic species and is more mobile than AsIII in both phloem and xylem transport (Carey et al., 2010). In addition, Carey et al. (2011) also found that inorganic As is poorly remobilized from flag leaves to grain through phloem transport, while DMA^V^ and MMA^V^ are efficiently retranslocated. Moreover, they also speculated that stem translocation of inorganic As may not rely solely on Si transporters (Carey et al., 2011). More recently, two inositol transporters (INT) responsible for arsenite uptake in the phloem in *Arabidopsis thaliana*, AtINT2 and AtINT4, have been identified. The disruption of AtINT2 or AtINT4 reduces As concentrations in phloem and seed in plants fed with AsIII through the roots or leaves, suggesting that inositol transporters may mediate AsIII loading into the phloem (Duan et al., 2015). However, whether there are similar transporters responsible for As transport in rice is still unknown.

## Breeding Rice for Low As

A simple method to decrease As in rice is to select cultivars that biologically restrict As accumulation in the grains as some rice cultivars accumulate 20–30 fold less As than others (Norton et al., 2012; Syu et al., 2015; Zhang et al., 2016). These cultivars may have developed ways of blocking As uptake, translocation or accumulation, providing gene resources to help breed low-As rice. While quantitative trait loci associated with As accumulation in rice have been recognized (Zhang et al., 2008; Norton et al., 2014), the candidate genes have not been confirmed. The genetic variability in rice As accumulation suggests that there could be additional germplasm for a low As uptake and accumulation trait in wild rice species. Future studies are therefore needed to explore whether different rice species or varieties differ in uptake, translocation and/or accumulation of AsIII, Si and/or P in rice grains.

### Arsenate Reduction to Arsenite in Plants

Former studies showed that ACR2 arsenate reductase, like AtACR2 in *Arabidopsis* and OsACR2.1 and OsACR2.2 in rice, may involve in AsV reduction (Dhankher et al., 2006; Duan et al., 2007) (**Table 1**). However, more recent evidence showed that canonical ACR2 arsenate reductase probably does not play a significant role in arsenate reduction (Liu et al., 2012; Chao et al., 2014) (**Table 1**). Instead, a novel arsenate reductase, different from canonical ACR2, is critical for AsV reduction and AsV tolerance in *Arabidopsis*, which is termed ARQ1 (Arsenate Reductase QTL1) (Sanchez-Bermejo et al., 2014) or HAC1 (High Arsenic Content 1) (Chao et al., 2014) (**Table 1**).

In *Arabidopsis*, HAC1 reduces AsV to AsIII in the outer cell layer of the roots, facilitating AsIII efflux out into the external environment (Chao et al., 2014). Plants lacking HAC1 lose their ability of AsV reduction, decreasing AsIII efflux and increasing As translocation to the shoots (Chao et al., 2014). In rice, OsHAC1;1 and OsHAC1;2 are responsible for AsV reduction (Shi et al., 2016) (**Table 1**). Overexpressing OsHAC1;1 or OsHAC1;2 significantly increases AsIII efflux into the external medium and decreases As accumulation in rice. When cultivated in paddy soil supplemented with an environmentally relevant dose of AsV and irrigated regularly with free drainage, the *OsHAC1;1* and *OsHAC1;2* overexpression lines have ∼20% lower grain As (Shi et al., 2016).

In addition, the glutaredoxin may also play a role in AsV reduction and regulating the cellular AsIII levels, though the mechanistic details for its function are yet to be resolved (Sundaram et al., 2008, 2009). PvGrx5, a glutathione-dependent oxidoreductase from As-hyperaccumulator *Pteris vittata*, decreases As accumulation in the leaves in transgenic *Arabidopsis* (Sundaram et al., 2009). More recently, two rice glutaredoxins, OsGrx_C7 and OsGrx_C2.1 have been proved to be important determinants of As-stress response and involved in lowering AsIII accumulation in *Arabidopsis* (Verma et al., 2016) (**Table 1**).

### Arsenite Efflux to External Environment

Plants can rapidly reduce AsV to AsIII in the roots, which could then be effluxed out into external medium (Xu et al., 2007; Chen Y. et al., 2013; Chen Y.S. et al., 2015; Han et al., 2016). Enhancing AsIII efflux by plant roots could be a potential strategy to decrease As accumulation in plants. Until now, the key membrane transporters responsible for AsIII efflux in plant roots have not been characterized. The aquaporin Lsi1 (OsNIP2;1), which mediates AsIII uptake and confers As accumulation in rice, also mediates AsIII efflux, contributing to 15–20% of the total As efflux (Zhao et al., 2010a). Other plant aquaporins, like AtNIP3;1, AtNIP5;1, AtNIP6;1 and AtNIP7;1 from *Arabidopsis*, OsNIP3;2 from *O. sativa*, LjNIP5;1 and LjNIP6;1 from *Lotus japonicas*, and PvTIP4;1 from *P. vittata*, also transport AsIII bi-directionally, which is a passive process with the flux direction depending on the concentration gradient (Bienert et al., 2008; Isayenkov and Maathuis, 2008; Xu et al., 2015; He et al., 2016). Thus, manipulating the expression of aquaporins via genetic engineering to enhance AsIII efflux will likely be complicated.

In yeast, AsIII is extruded into the external environment by the AsIII efflux transporter ACR3 (Arsenic Compounds Resistance protein 3) (Wysocki et al., 1997). Interestingly, ACR3 is lost in flowering plants including rice, but exists in As-hyperaccumulator *P. vittata* with duplication (Indriolo et al., 2010). However, whether ACR3s are involved in AsIII efflux to external environment in *P. vittata* remains unclear.

As-hyperaccumulator *P. vittata* is extremely efficient in extracting As from soils and translocating it into the fronds, which can exceed 2.3% of its dry biomass (Ma et al., 2001). To help decrease As accumulation in rice, it is of interest to understand why hyperaccumulators are so efficient in accumulating As. In this aspect, *P. vittata* is characterized with limited AsIII complexation in the roots, limited AsIII efflux to the external medium but efficient xylem loading of AsIII to the fronds (Su et al., 2008). Recent report also shows that high As exposure induces substantial AsIII efflux in *P. vittata*, which may help the plant to alleviate As toxicity (Chen et al., 2016).

The fact that an ACR3 from *P. vittata*, PvACR3, localizes to the vacuolar membrane indicates that it likely extrudes AsIII into the vacuoles for sequestration (Indriolo et al., 2010). However, in transgenic *Arabidopsis*, PvACR3 localizes to the plasma membrane and significantly increases AsIII efflux, thereby decreasing As accumulation by ∼90% in the roots compared to that of wild-type (Chen Y. et al., 2013). Meanwhile, the transgenic plants accumulate more As in the shoots after long-term cultivation in soils, probably because PvACR3 confers AsIII efflux toward or into xylem for translocation in root stele cells and extrudes AsIII into apoplast for sequestration in leaf cells (Chen Y. et al., 2013). Unlike aquaporins dependent on the concentration gradient, AsIII efflux transporter ACR3 functions via the proton motive force for energy (Wysocki et al., 1997), and hence may be an ideal candidate gene to enhance AsIII efflux and decrease As accumulation in rice (**Table 1**).

After introducing yeast *ACR3* (*ScACR3*) into rice, the transgenic plant exhibits higher As efflux by the roots, lowering As accumulation in rice grains by ∼20% (Duan et al., 2012). Another *P. vittata* ACR3, PvACR3;1, has not been well characterized so its function remains unknown. It is speculated that when ACR3 localizes in plant root endodermis or xylem parenchyma cells, it may also mediate AsIII efflux into the xylem for translocation (Ali et al., 2012; Chen Y. et al., 2013), similar to the effects of Lsi2 in rice (Ma et al., 2008). To exert the AsIII efflux functions of ACR3 and reduce additional AsIII translocation, a root exodermis specific ACR3 expression should be tested in transgenic rice.

### Arsenic Sequestration in Vacuoles

In plants, AsIII-PC can be sequestrated into vacuoles as a step of As detoxification in cells, which also affects As allocation in plant tissues. In rice, OsABCC1 mediates vacuolar AsIII–PC sequestration, thus reducing As accumulation in rice grains (Song et al., 2014). In the roots, OsABCC1 is expressed in the exodermis and pericycle (**Figure 1**). However, *Osabcc1* mutant does not show decreased As accumulation in the roots compared with wild-type rice at relatively low As concentrations, probably because knockout of *OsABCC1* results in increased toxicity, inducing the biosynthesis of thiol compounds that bind to As in cytoplasm (Song et al., 2014). In the shoots, knockout of OsABCC1 decreases As accumulation in node I, but increases As allocation to the flag leaf and grain, leading to 13- to 18-fold higher As in brown rice than those of wild-type (Song et al., 2014). Thus, overexpression of OsABCC1 may be useful to breed low-As rice.

Overexpressing transporters for As sequestration in the shoots may lead to As accumulation in plants (Song et al., 2003; Zhu and Rosen, 2009; Guo et al., 2012). However, overexpression of relevant genes in the roots may decrease As accumulation in the shoots (Zhu and Rosen, 2009). Vacuolar sequestration in the roots can function as a barrier to limit metal translocation to the shoots (Ueno et al., 2010). The presence of ABC transporters, including YCF1, AtABCC1/2 and OsABCC1, suggests that this strategy can be applied in rice to decrease As accumulation. Because complexation of AsIII by thiols is a critical step for As transport into the vacuoles, simultaneously expressing the ABC transporters and PC synthase, the rate-limiting step in PC biosynthesis, may maximize As sequestration. In addition, root-specific promoters may be useful in controlling gene expression for genetically engineering low-As rice.

### Arsenic Methylation and Volatilization

Though As methylation is widespread in bacteria, fungi, algae, animals and humans, probably as a detoxification process, As methylation *in vivo* in plants is unknown (Bentley and Chasteen, 2002; Gebel, 2002; Qin et al., 2006). By examining microbial genomes, Qin et al. (2006) identified a gene encoding an AsIII *S*-adenosylmethionine methyltransferase (ArsM) from the bacterium *Rhodopseudomonas palustris* (**Table 1**). They showed that RpArsM catalyzes the formation of a number of methylated intermediates (DMA^V^ and TMAO) from AsIII, with TMAs^III^ gas as the final product. In addition, Qin et al. (2009) identified two ArsM from the eukaryotic alga *Cyanidioschyzon merolae* (**Table 1**).

Mammalian AS3MT is homologous to bacterial ArsM (Qin et al., 2006; Ye et al., 2012; Chen J. et al., 2013). However, to date, corresponding enzymes for As methylation with significant homology to ArsM/AS3MT in higher plants have not been detected (Ye et al., 2012). Although a gene from rice (*Os02g51030*) contains similar motif with ArsM/AS3MT (Norton et al., 2008), it does not contain the core region of ArsM/AS3MT, critical for methyl group transfer to As (Ye et al., 2012).

Expression of ArsM gene in rice may catalyze As methylation and volatilization, thus providing a strategy to reduce accumulation of toxic As species and/or total As in rice grains. Meng et al. (2011) transformed the RpArsM gene into rice and found the transgenic rice produces methylated As species and gives off 10-fold greater volatile arsenicals compared to the control. The results also show that As accumulation decreases in T1 generation transgenic rice grains including AsIII and AsV concentrations. However, in this study the volatile arsenicals account for only 0.06% of the total As in plants. Therefore, to introduce As methylation into rice, optimization of heterologous gene expression and regulation is necessary. More recently, Tang et al. (2016) genetically engineered *A. thaliana* with *ArsM* from the eukaryotic alga *Chlamydomonas reinhardtii*. They found the transgenic plants methylate most of the inorganic As to DMA^V^ in the shoots, exhibiting higher phytotoxicity than inorganic As in *Arabidopsis*.

## Application of Gene-Editing

Although critical genes responsible for As uptake, transport and detoxification can reduce As accumulation in rice grains, limited natural genetic resources may ultimately restrict their application. In this context, gene-editing technologies are of great interest to both gene function characterization and crop improvement. The RNA-guided CRISPR/Cas9 system, which depends on bacterial Cluster Regularly Interspaced Short Palindromic Repeats (CRISPR)-associated nuclease (Cas), is emerging as the tool of choice for precise gene editing (Cong et al., 2013; Ran et al., 2013). Different from other gene editing technologies like Zinc-Finger Nucleases (ZFNs) and Transcription Activator–Like Effector Nucleases (TALENs), RNA-guided CRISPR/Cas9 system is easy to design, has high specificity, and is well-suited for high-throughput and multiplexed gene editing for a variety of organisms including rice (Ran et al., 2013; Shan et al., 2013; Xie and Yang, 2013; Schiml et al., 2014; Zhang et al., 2014; Ma et al., 2015).

**FIGURE 2:**
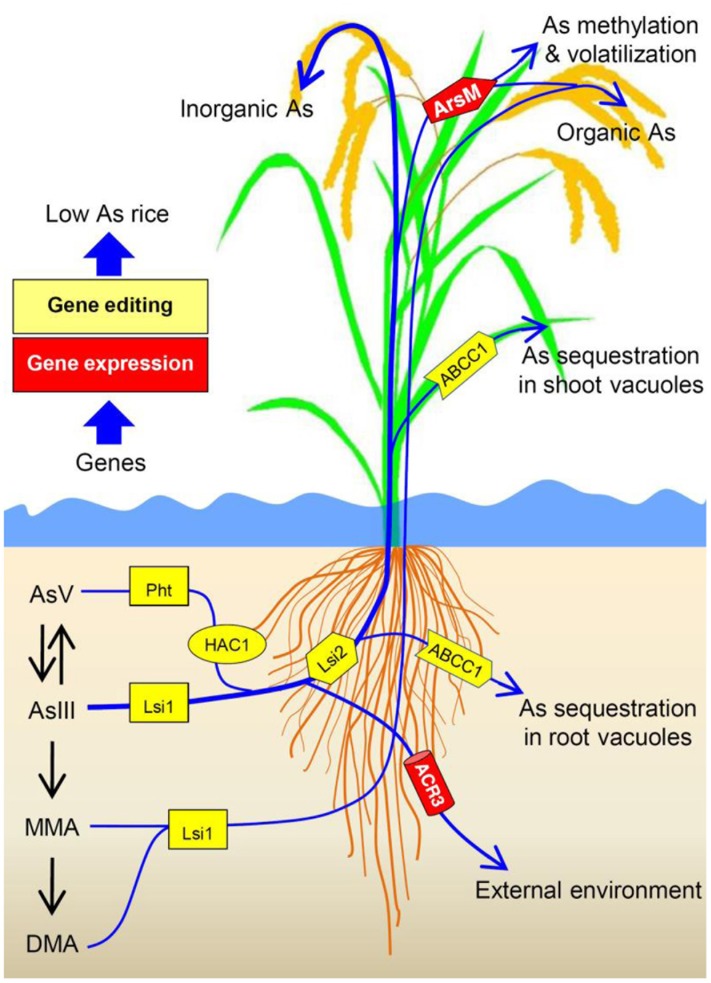
**Schematic diagram showing arsenic (As) uptake pathway and the endogenous (Pht, Lsi1, ACR2, HAC1, Lsi2 NRAMP1 and ABCC1 genes from rice) and exogenous genes (ArsM and ACR3 genes from other species) that can be manipulated to decrease As transport to and accumulation in rice grains.** Rice phosphate transporters (Pht) and aquaporin Lsi1 contribute to As uptake. Arsenate reductase HAC1 is responsible for arsenate (AsV) reduction in rice. Lsi2 plays a critical role in As transport toward root xylem for As translocation, thus promotes As transport to and accumulation in rice grains. NRAMP1 assists in As transport to root xylem. In contrast, ABCC1 mediates As sequestration in vacuoles, especially in rice roots and nodes, restricting As transport to grains. Heterologous expression of *ACR3* may enhance arsenite (AsIII) efflux while expressing *ArsM* may confer As methylation and subsequent volatilization. Transgenic approach and/or gene editing can be used to manipulate the targeted genes to produce low-As rice.

To produce low-As rice, critical genes that are responsible for As uptake and transport (e.g., *OsPht1:8. Lsi1* and *Lsi2*) might be early targets for gene editing (**Figure 2**). While engineering rice with CRISPR/Cas9 for mutations in *OsPht1:8* and *Lsi1/2* could be a strategy for reducing As uptake by rice, such manipulation may also influence P and Si uptake. Thus, one could search for allelic variations in *OsPht1:8* and *Lsi1/2* that could selectively transport P and Si over AsV and AsIII, thereby reducing As uptake. In addition, endogenous *OsNRAMP1* and *OsABCC1* genes in rice could also be selected as targets for CRISPR/Cas9-based disruption or modification to develop low-As rice.

## Concluding Remarks and Future Interests

Reducing the levels of the ubiquitous carcinogenic As in rice is a major public health goal. Arsenic levels and species vary widely in paddy soils for different regions and within different rice cultivars. During the past decade, molecular biology research on how plants deal with As has opened up unprecedented opportunities to make the rice grains safer by lowering its As content. Research using transgenic systems can inform plant breeders to select certain genetic markers over others to obtain low-As rice varieties. In addition, newly developed gene-editing technology can also help in altering endogenous genes (**Figure 2**). It is important to elucidate how rice and other plant species metabolize As so that new genes can become available for further improvement to produce low-As rice.

## Author Contributions

YCh wrote the article, prepared the illustrations and incorporated edits from co-authors, and approved the final draft. Y-HH, YCa, and Y-GZ provided intellectual content and editorial suggestions for the manuscript. BR and LM conceptualized the overall structure of the review article, critically edited it and approved the final draft.

## Conflict of Interest Statement

The authors declare that the research was conducted in the absence of any commercial or financial relationships that could be construed as a potential conflict of interest.

## Footnotes

1 http://faostat3.fao.org/
